# Immune-related thyroid dysfunction as a positive prognostic factor for patients with lung cancer in China: a real-world retrospective study

**DOI:** 10.3389/fimmu.2024.1495460

**Published:** 2024-12-23

**Authors:** Ge Guo, Zihan Jing, Wenrui Dou, Guiqin Wang, JunJie Dang, Yajie Li, Ruqiong Wang, Huan Zhang, Jing Sun, Lihua Shang

**Affiliations:** ^1^ Department of Medical Oncology, Harbin Medical University Cancer Hospital, Harbin, China; ^2^ Department of Clinical Medicine, Harbin Medical University, Harbin, China; ^3^ School of Marxism, Sanquan College of Xinxiang Medical University, Xinxiang, China

**Keywords:** lung neoplasms, immune checkpoint inhibitor, irAEs, immune-related thyroid dysfunction, prediction biomarker

## Abstract

**Introduction:**

The relationship between immune-related thyroid dysfunction (irTD) and survival rates in cancer patients remains unclear. Furthermore, the impact of variations in immunotherapy line numbers and pathological types among lung cancer patients on this relationship has not been fully elucidated. This study aims to evaluate the potential of irTD as a prognostic marker for immunotherapy in Chinese patients with lung cancer.

**Methods:**

A retrospective analysis was conducted on data collected from patients with locally advanced or metastatic lung cancer who received immune checkpoint inhibitor treatment at the Harbin Medical University Cancer Hospital. The study period spanned from December 1, 2016, to November 30, 2023. The primary endpoints were progression-free survival (PFS) and overall survival (OS), while the objective response rate served as the secondary endpoint.

**Results:**

Among the 361 patients in this study, 42.7% developed irTD. Significant differences were observed between the groups with and without irTD regarding inflammatory indices, thyroid-stimulating hormone levels, and thyroid autoantibody positivity (P < 0.05). Patients with irTD demonstrated longer OS (32.5 vs. 22 months, HR: 0.65, 95% CI: 0.49-0.88; P = 0.005). For NSCLC patients, OS was significantly prolonged in those with irTD (40.8 vs. 27.2 months, HR: 0.68, 95% CI: 0.48-0.96; P = 0.028). Similarly, SCLC patients who developed irTD exhibited longer OS (27.9 vs. 13.8 months, HR: 0.51, 95% CI: 0.29-0.90; P = 0.022). Notably, irTD was observed exclusively in patients receiving immunotherapy in the second or later lines, showing a significant association with extended OS (40.8 vs. 19.4 months, HR: 0.56, 95% CI: 0.35-0.88; P = 0.012), while the presence of irTD during first-line immunotherapy did not confer a benefit to patients (32.4 vs 24.5 months, HR: 0.74, 95% CI: 0.50-1.10; P = 0.134). The effects of different irTD types, severities, or clinical symptoms on PFS and OS did not differ significantly (P > 0.05).

**Conclusion:**

irTD demonstrates potential as a predictive marker for long-term survival benefits in Chinese patients with lung cancer. However, our exploratory analysis indicates that this association was exclusively observed in individuals receiving immunotherapy as a second-line or subsequent treatment.

## 1 Introduction

Immune checkpoint inhibitor (ICI) therapy, a novel class of anti-tumor medication, has become integral to cancer treatment due to their remarkable efficacy against various malignancies (1, 2). Unlike conventional chemotherapy, ICIs stimulate the body’s immune system to elicit antitumor activity (3). However, this activation can occasionally result in immune-related adverse effects (irAEs) affecting any organ at any stage of treatment (4). Immune-related thyroid dysfunction (irTD), with an incidence rate of up to 40%, is the most common irAE impacting the endocrine system (5). Patients experiencing irTD often exhibit symptoms such as fatigue, weight fluctuations, and emotional instability, which can compromise their quality of life and tolerance to anti-tumor treatments, potentially reducing treatment adherence. Mild thyroid dysfunction typically necessitates only close monitoring without immediate intervention. However, if a patient develops significant symptoms or substantial thyroid dysfunction, such as severe thyrotoxicosis or hypothyroidism, medication becomes necessary. Patients with hypothyroidism may require thyroid hormone replacement therapy, while those with thyrotoxicosis may need antithyroid drugs (6). This additional treatment burden on patients, particularly in cases of severe thyroid dysfunction, may necessitate long-term monitoring and management. Consequently, the high incidence of irTD has raised considerable concern among clinicians regarding its potential adverse effects on patients.

Multiple studies have suggested that the occurrence of irTD is associated with enhanced immune responses and survival benefits. However, the findings are not universally consistent. For instance, a retrospective analysis of 48 NSCLC patients in the KEYNOTE-001 study demonstrated that those receiving pembrolizumab who developed irTD experienced significantly longer OS (HR = 0.29; 95% CI: 0.09-0.94; P = 0.04) (7). A recent Chinese study corroborated irTD as a valuable prognostic indicator, revealing a significant correlation between irTD and extended OS (HR = 0.67, 95% CI: 0.45-0.99; P = 0.046) and PFS (HR =0.61, 95% CI: 0.44-0.86; P = 0.005) (8). In an evaluation of 58 patients with stage IV NSCLC to assess the short-term efficacy of immunotherapy with PD-1 inhibitors, patients exhibiting irTD showed higher rates of disease control (15.8% vs. 0.0%, P = 0.011) and objective response (31.6% vs. 10.3%, P = 0.044) (9). Nevertheless, the efficacy of irTD in relation to immunotherapy remains inconclusive. Numerous studies have reported varied outcomes regarding the impact of irTD on treatment results. This variability highlights the complexity of the relationship between irTD and immunotherapy efficacy. A large-scale study involving 1,246 patients, with a median follow-up of 11.3 months, found no significant difference in mortality between patients with thyroid irAEs and those without (33% vs. 37%; P = 0.14). Furthermore, no difference in PFS or OS was observed in patients with overt hypothyroidism (10). Nervo A et al. also reported no statistical differences in PFS between patients with and without irTD (11). These findings align with other research that questions the direct impact of irTD on treatment outcomes, suggesting that irTD may not be a reliable predictor of PFS and OS in patients undergoing immunotherapy. Therefore, more comprehensive research is necessary to elucidate the relationship between irTD and survival benefit.

In recent years, it has been realized that determining the optimal initiation time for immunotherapy is crucial for clinical decision-making. For NSCLC, the selection of first-line treatment is contingent upon the presence or absence of driver gene mutations. For patients with driver gene-positive NSCLC (such as EGFR, ALK, or ROS1 mutations), targeted therapies, including EGFR inhibitors or ALK inhibitors, are the standard of care. Conversely, for patients with driver gene-negative NSCLC, treatment typically involves immunotherapy based on PD-1/PD-L1 inhibitors, often combined with chemotherapy. In second-line therapy, the combination of anti-angiogenic agents (e.g., Anlotinib) and immune checkpoint inhibitors has emerged as a common approach. For extensive-stage small cell lung cancer (SCLC), the first-line treatment usually consists of chemotherapy combined with immunotherapy, such as PD-L1 inhibitors (Atezolizumab), while second-line treatment relies more on single-agent chemotherapy or single-agent immunotherapy. A retrospective study analyzed 126 patients with NSCLC treated with Pembrolizumab, Sintilimab, Atezolizumab, or Camrelizumab as first-line therapy. Despite the demonstrated clinical benefits of these drugs across various tumor types, no statistically significant improvements in progression-free survival (PFS) or overall survival (OS) were observed in patients with irTD compared to those with normal thyroid function (12). This finding has prompted reconsideration of the potential influence of irTD in the context of immunotherapy, particularly regarding whether the timing of treatment initiation might modify its effects. No studies examining the impact of irAE or irTD have been conducted exclusively in patients receiving immunotherapy as the second or later line of treatment. The immune system’s initial response in second-line or later therapies may differ from that in first-line treatments, potentially altering the prognostic significance of irTD across different lines of immunotherapy.

This study sought to investigate the impact of irTD on the prognosis of lung cancer patients undergoing immunotherapy in China. By examining the potential influence of diverse pathologic types and treatment lines on the relationship between irTD occurrence and immunotherapy response, the research aims to provide clinicians with crucial clinical data and insights. The findings will contribute to assessing the predictive role of irTD in various immunotherapy populations and facilitate the development of more precise and personalized treatment strategies.

## 2 Materials and methods

### 2.1 Patients enrollment

**Figure 1:**
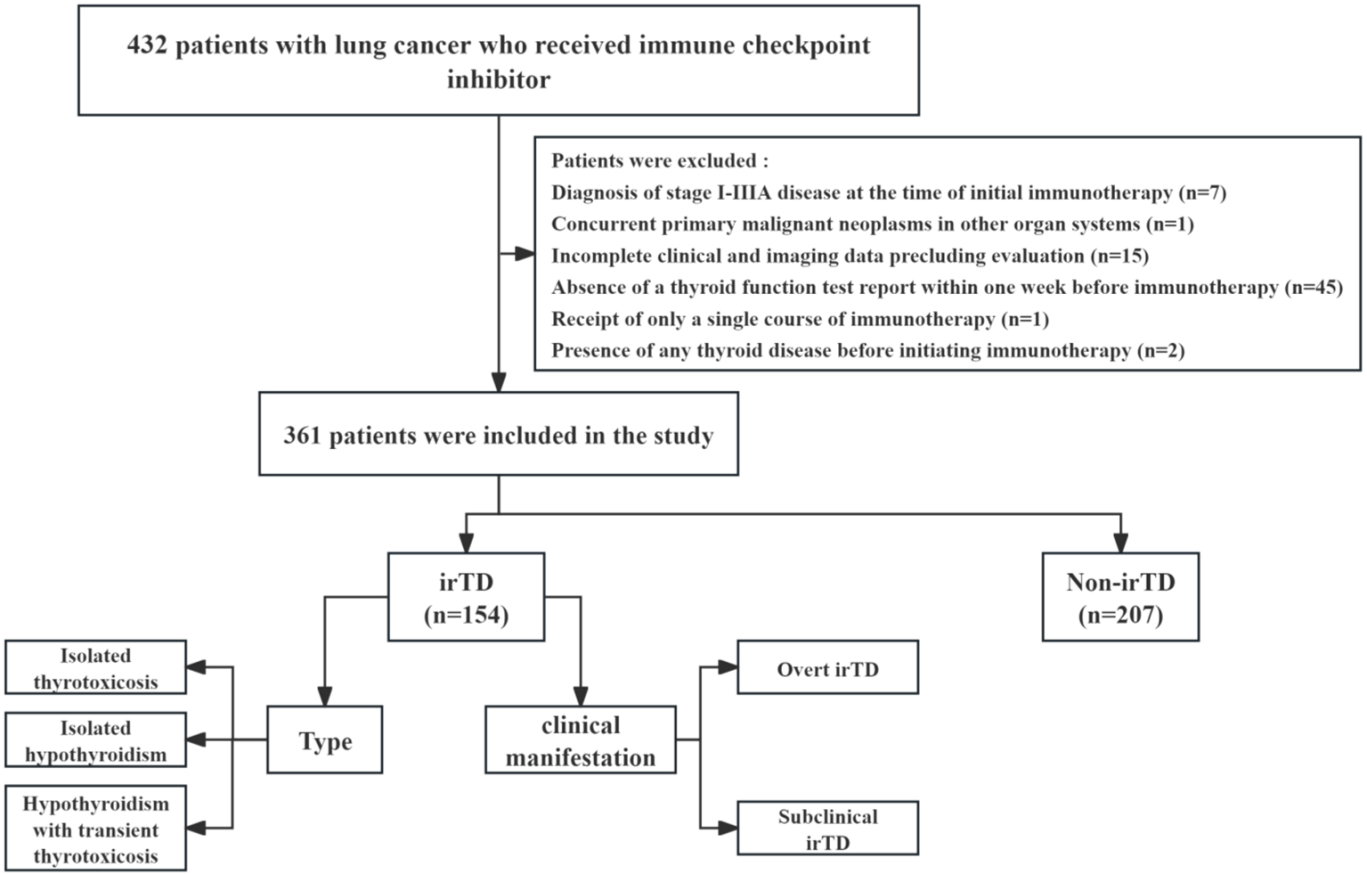
Schematic representation of the patient inclusion process.

A review of the clinical data database at Harbin Medical University Cancer Hospital identified patients with locally advanced or metastatic lung cancer who received immune checkpoint inhibitors between December 1, 2016, and November 30, 2023. These individuals were retrospectively included in the study, and **Figure 1** illustrates the comprehensive screening process. The inclusion criteria were: (1) Histologically confirmed diagnosis of NSCLC or SCLC; (2) Clinician’s assessment of locally advanced or metastatic disease, according to the eighth edition of the Metastatic Tumor Staging System; (3) Receipt of at least two courses of immunotherapy and two thyroid function tests following the initial immunotherapy; (4) Hematological tests conducted at this institution within one week prior to immunotherapy administration. The exclusion criteria were: (1) Presence of any thyroid disease before initiating immunotherapy; (2) Absence of a thyroid function test report within one week before immunotherapy; (3) Incomplete clinical and imaging data precluding evaluation; (4) Receipt of only a single course of immunotherapy; (5) Diagnosis of stage I-IIIA disease at the time of initial immunotherapy; (6) Concurrent primary malignant neoplasms in other organ systems.

The collected patient data encompassed demographic characteristics (gender, age, and smoking history), clinical characteristics (ECOG performance status, tumor stage, and tumor type), and the number of immunotherapy lines administered. Laboratory data from peripheral blood samples were obtained prior to the initiation of immunotherapy.

This study adhered to the principles outlined in the Declaration of Helsinki. The Ethics Committee of Harbin Medical University Cancer Hospital granted approval for the research on January 14, 2022 (No. KY2021-47).

### 2.2 Groupings and definitions

Patients with irTD may present with either thyrotoxicosis or hypothyroidism in overt or subclinical forms. The study participants were divided into two groups: irTD and non-irTD. The irTD group was further categorized into three subgroups based on the type of irTD: (1) isolated thyrotoxicosis, (2) isolated hypothyroidism, and (3) hypothyroidism with transient thyrotoxicosis. Additionally, patients with irTD were classified as either overt or subclinical based on their clinical symptoms. The onset of irTD was determined by calculating the time interval between the first administration of immunotherapy and the manifestation of irTD symptoms. Thyroid autoantibody positivity was defined as TGAb levels exceeding 115 IU/ml or TMAb levels exceeding 34 IU/ml prior to immunotherapy initiation. Changes in thyroid autoantibody levels during immunotherapy were also documented. Newly developed antibody positivity was defined as baseline antibody levels within the normal reference range and post-treatment TGAb or TMAb levels above the upper limit of the reference range. A significant increase in antibody was defined as a positive baseline TGAb or TMAb with a > 50% increase in antibody titers after treatment. The neutrophil-to-lymphocyte ratio (NLR) was calculated as the ratio of neutrophil count to lymphocyte count, while the monocyte-to-lymphocyte ratio (MLR) was defined as the ratio of monocyte count to lymphocyte count.

### 2.3 Outcome assessment

The primary endpoints of the study were PFS and OS, while the ORR served as a secondary endpoint. Efficacy assessment was conducted using the RECIST v1.1. PFS and OS were calculated for each participant. PFS was defined as the time from the initiation of immunotherapy to disease progression, death from any cause, or the follow-up cut-off date. OS was measured from the start of immunotherapy treatment to death from any cause or the follow-up cut-off date. The ORR was determined as the percentage of patients achieving a tumor volume reduction of at least 30%, sustained for a minimum of four weeks.

### 2.4 Statistical analyses

The data were presented as total case numbers and percentages (%). Group comparisons utilized the chi-square test (χ² test) or Fisher’s exact probability method. For non-normally distributed variables, the median (M) and interquartile range (Q1, Q3) were reported. The Mann-Whitney U test was employed for group comparisons of these variables. PFS and OS curves were plotted using the Kaplan-Meier method. Survival statistics were compared using the log-rank test, with a two-tailed P value < 0.05 considered statistically significant. Landmark analysis was applied when survival curves intersected. Variables showing statistically significant correlations underwent initial univariate analysis. These variables were subsequently included in a multivariate logistic regression model, utilizing the input method with a significance level of α = 0.05. Statistical analyses were conducted using three software tools: R 4.3.2, GraphPad Prism 10.1.1, and SPSS 25.0.

## 3 Result

### 3.1 Patient characteristics

The study included 361 patients with a median follow-up duration of 33.9 months. As shown in **Table 1**, 246 patients (68.1%) were male, and 226 (62.6%) were under 65 years of age. Furthermore, 213 patients (59.0%) had a body mass index (BMI) below 24.0, and 190 patients (52.6%) had a history of smoking. The majority of patients were in good physical condition, with 335 (92.8%) having an ECOG score below 2. Among the total cohort, 288 (79.8%) had NSCLC, and 222 (61.5%) received immunotherapy as first-line treatment. In the NSCLC cohort, 95.1% of patients received PD-1 inhibitor treatment, with only one stage IV patient receiving a combination of CTLA-4 and PD-1 inhibitors. In the SCLC cohort, 76.7% of patients were treated with durvalumab, a PD-L1 inhibitor.

**Table 1:** Characteristics of enrolled patients in all, irTD group and non-irTD group.

Variables	Total (n=361)	irTD (n=154)	Non-irTD (n=207)	P-Value
**Age, n (%)**				0.100
≥65	135 (37.4)	50 (32.5)	85 (41.1)	
<65	226 (62.6)	104 (67.5)	122 (58.9)	
**Gender, n (%)**				0.304
Male	246 (68.1)	100 (64.9)	146 (70.5)	
Female	115 (31.9)	54 (35.1)	61 (29.5)	
**BMI, n (%)**				0.703
<24	213 (59.0)	89 (57.8)	124 (59.9)	
24-27.9	118 (32.7)	50 (32.5)	68 (32.9)	
≥28	30 (8.3)	15 (9.7)	15 (7.2)	
**Smoking, n (%)**				0.059
No	190 (52.6)	79 (51.3)	111 (53.6)	
Yes	171 (47.4)	75 (48.7)	96 (46.4)	
**ECOG score, n (%)**				0.148
<2	335 (92.8)	147 (95.4)	188 (90.8)	
≥2	26 (7.2)	7 (4.6)	19 (9.2)	
**Tumor types, n (%)**				0.792
NSCLC	288 (79.8)	124 (80.5)	164 (79.2)	
SCLC	73 (20.2)	30 (19.5)	43 (20.8)	
**Line of immunotherapy, n (%)**				0.190
1	222 (61.5)	101 (65.6)	121 (58.5)	
≥2	139 (38.5)	53 (34.4)	86 (41.5)	
**Baseline MONO** **(×10^9^/L)**	0.52 (0.38-0.66)	0.49 (0.35-0.62)	0.55 (0.41-0.68)	**0.004**
**Baseline NE** **(×10^9^/L)**	4.53 (3.42-5.94)	4.51 (3.29-5.73)	4.56 (3.44-6.10)	0.299
**Baseline Lym** **(×10^9^/L)**	1.61 (1.20-2.02)	1.65 (1.29-2.01)	1.57 (1.15-2.09)	0.299
**Baseline NLR**	2.92 (1.93-4.24)	2.67 (1.83-3.86)	3.05 (2.02-4.57)	**0.033**
**Baseline MLR**	0.32 (0.23-0.44)	0.30 (0.21-0.41)	0.34 (0.24-0.47)	**0.004**
**Baseline TSH (µIU/ml)**	1.88 (1.31-3.24)	2.50 (1.58-3.92)	1.72 (1.20-2.24)	**<0.001**
**Baseline TGAb, n (%)**				**<0.001**
Negative	273 (75.6)	107 (69.5)	166 (80.2)	
Positive	20 (5.5)	18 (11.6)	2 (0.9)	
Unidentified	68 (18.9)	29 (18.9)	39 (18.9)	
**Baseline TMAb, n (%)**				**0.002**
Negative	277 (76.7)	112 (72.7)	165 (79.7)	
Positive	16 (4.4)	13 (8.4)	3 (1.4)	
Unidentified	68 (18.9)	29 (18.9)	39 (18.9)	
Changes thyroid autoantibodies in immunotherapy, n (%)				
Newly developed antibody-positive	33 (9.1)	25 (16.2)	8 (3.9)	
Significant increase in antibody	17 (4.7)	13 (8.4)	4 (1.8)	
Other (uncertain and antibody negative)	311 (86.2)	116 (75.4)	195 (94.3)	

irTD, immune-related thyroid dysfunction; MONO, mononuclear cells; NE, neutrophils; Lym, lymphocytes; NLR, neutrophil-to-lymphocyte ratio; MLR, monocyte-to-lymphocyte ratio; TSH, thyroid-stimulating hormone; TGAb, anti-thyroglobulin antibodies; TMAb, anti-thyroid microsomal antibodies.

The bolded values indicate that the data are statistically significant (p < 0.05).

irTD was observed in 154 patients (42.7%). As shown in **Table 1**, no significant differences were observed between the irTD and non-irTD groups regarding age, gender, BMI, ECOG score, tumor type, and the number of immunotherapy lines. However, patients in the irTD group exhibited lower baseline monocyte levels (MONO: 0.49 [0.35-0.62] vs. 0.55 [0.41-0.68], P = 0.004) and lower NLR: 2.67 [1.57-4.57] vs. 3.05 [2.02-4.57], P = 0.033) compared to the non-irTD group. The MLR was also significantly lower in the irTD group (0.30 [0.21-0.41] vs. 0.34 [0.24-0.47], P = 0.004). Furthermore, patients in the irTD group demonstrated significantly higher baseline TSH levels compared to those in the non-irTD group (2.50 [1.58-3.92] vs. 1.72 [1.20-2.24], P < 0.001).

A total of 293 patients underwent testing for TGAb and TMAb; their data is presented in **Table 1**. The results indicate significantly higher positive rates of TGAb and TMAb in the irTD group compared to the non-irTD group (TGAb: 11.6% vs. 0.9%, P < 0.001; TMAb: 8.4% vs. 1.4%, P = 0.002). Baseline TGAb positivity demonstrated a specificity of 98.8% (166/168) and a sensitivity of 14.4% (18/125) for predicting irTD occurrence. Similarly, baseline TMAb positivity showed a specificity of 98.2% (165/168) and a sensitivity of 10.4% (13/125). Furthermore, the study monitored changes in thyroid autoantibody levels during immunotherapy. The findings revealed that 50 patients tested positive for thyroid autoantibodies throughout the treatment, comprising 33 cases of newly developed antibody-positivity and 17 cases of significant antibody increase. The irTD group exhibited notably higher incidence rates of newly developed antibody-positivity (16.2% vs. 3.9%) and significant antibody increase (8.4% vs. 1.8%) compared to the non-irTD group (**Table 1**).

### 3.2 An analysis of the various irTDs

**Figure 2:**
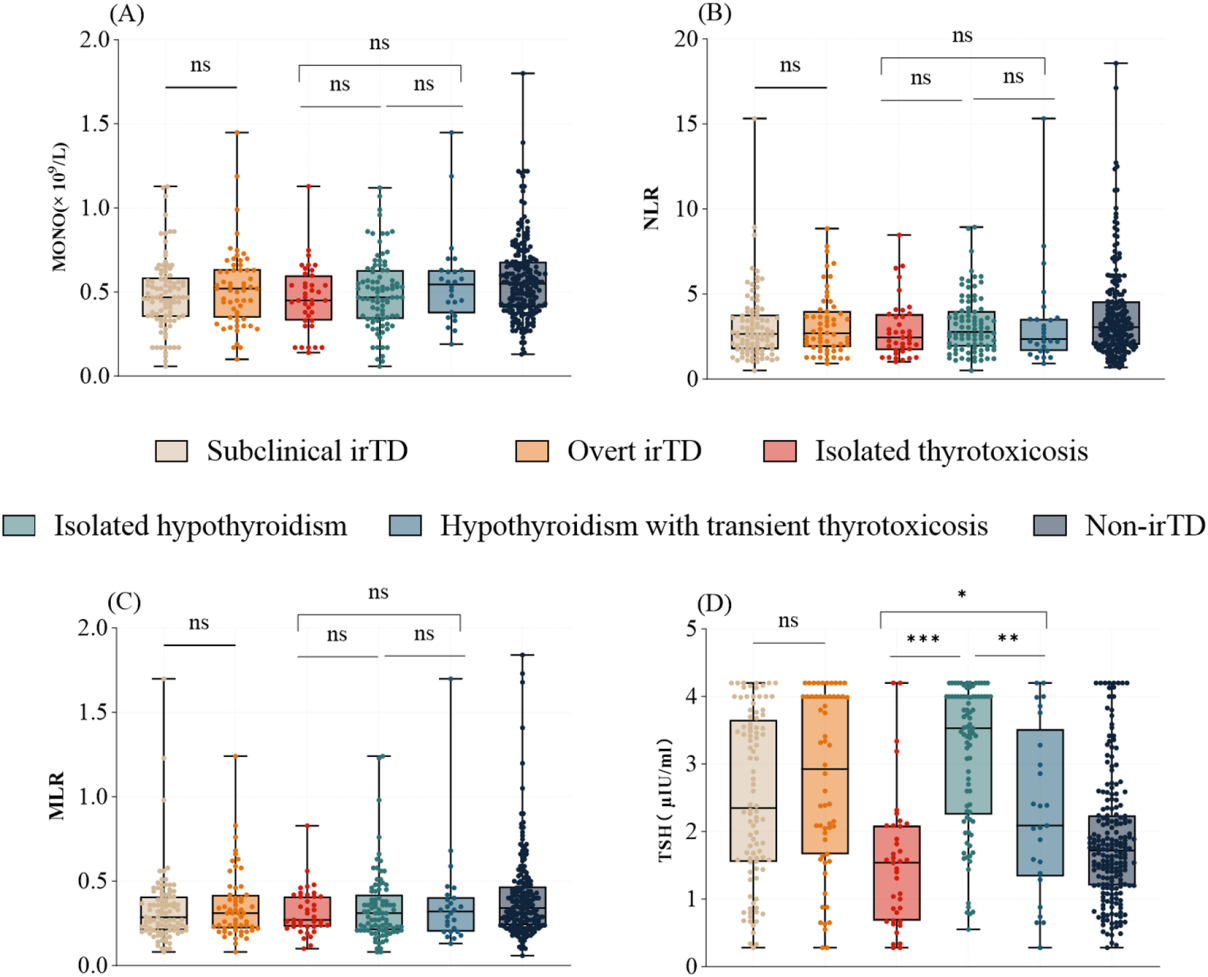
The distribution of MONO **(A)**, NLR **(B)**, MLR **(C)**, and TSH **(D)** varies with clinical presentation and type of irTD. *: 0.01 < p ≤ 0.05; **: 0.001 < p ≤ 0.01; ***: p ≤ 0.001; Not significant (ns): P > 0.05.

The distribution of inflammatory markers, including MONO, NLR, and MLR, showed no significant differences across various clinical presentations and types of irTD (**Figures 2A–C**). However, patients with isolated hypothyroidism exhibited significantly higher TSH levels compared to those with isolated thyrotoxicosis or hypothyroidism with transient thyrotoxicosis (3.53 [2.25-4.00] vs. 1.54 [0.68-2.09], P < 0.001; 3.53 [2.25-4.00] vs. 2.09 [1.34-3.52], P = 0.002) (**Figure 2D**).

**Figure 3:**
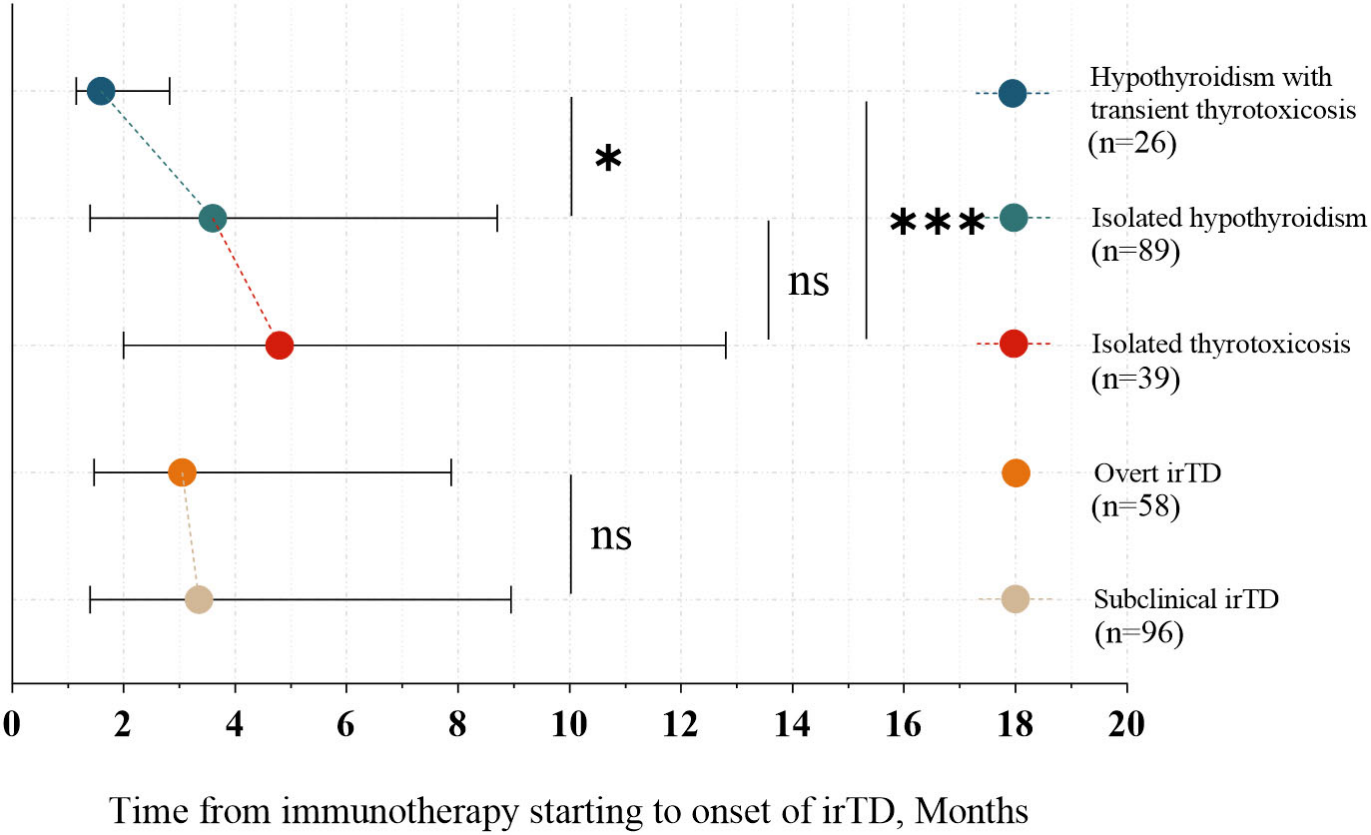
Onset time of different clinical presentations and types of irTD. *: 0.01 < p ≤ 0.05; ***: p ≤ 0.001; Not significant (ns): P > 0.05.

In 83% of patients, irTD manifested within the first year of immunotherapy. The time to onset did not differ significantly between the overt and subclinical irTD groups (3.05 months [1.48-7.88] vs. 3.35 months [1.40-8.95]; P = 0.962) (**Figure 3**). However, patients who developed hypothyroidism following transient thyrotoxicosis experienced a significantly earlier onset compared to those with isolated hypothyroidism and isolated thyrotoxicosis (1.60 months [1.15-2.83] vs. 4.80 months [2.00-12.80], P = 0.0008; 1.60 months [1.15-2.83] vs. 3.60 months [1.40-8.70], P = 0.018) (**Figure 3**).

### 3.3 Correlation between outcome, prognosis, and irTD

**Figure 4:**
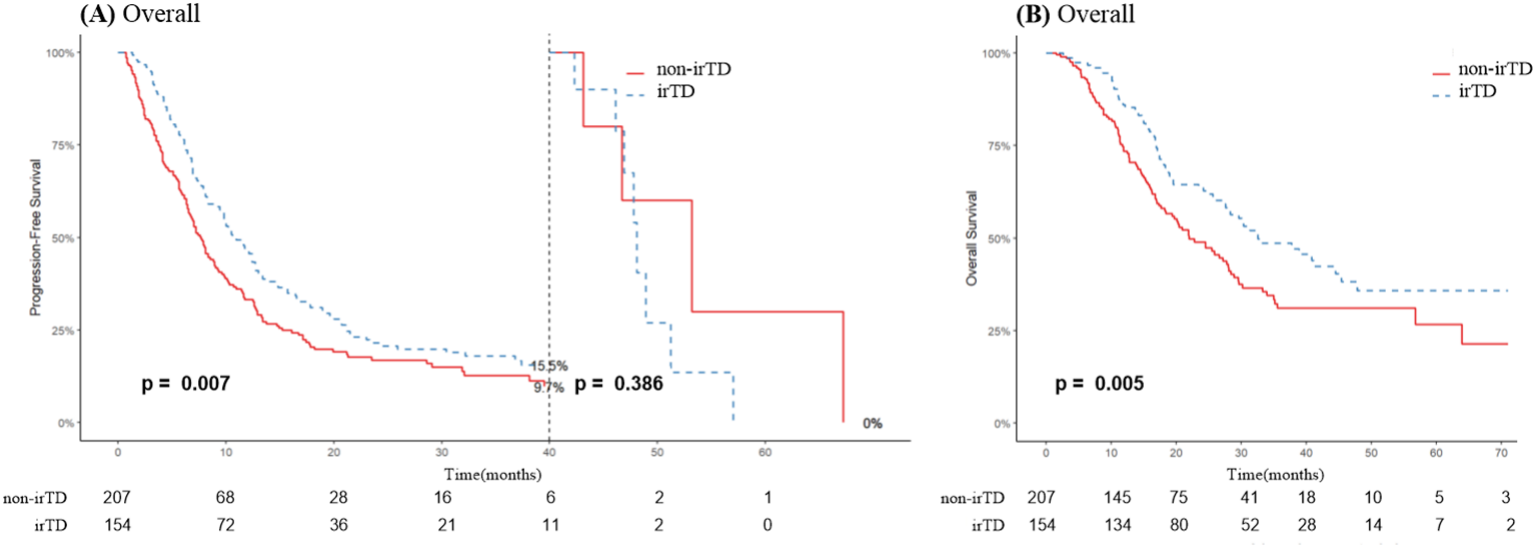
PFS **(A)** and OS **(B)** curves of patients with lung cancer with irTD vs non-irTD groups.

As illustrated in **Figure 4A**, irTD demonstrated significant efficacy in lung cancer patients, preventing disease progression for up to 40 months, after which its effect appeared to diminish. Notably, the median overall survival (mOS) of the irTD group was 32.5 months, compared to 22 months for the non-irTD group (HR: 0.65, 95% CI: 0.49-0.88; P = 0.005) (**Figure 4B**). Multivariate Cox regression analysis confirmed the sustained benefits of the irTD group in both PFS (P = 0.016) and OS (P = 0.006) (**Table 2**).

**Table 2:** Multivariate Cox analysis results of PFS and OS.

Variables	Progression-free Survival		Overall Survival	
	HR (95CI)	P	HR (95CI)	P
With irTD				
No	1		1	
Yes	0.75 (0.59-0.95)	**0.016**	0.65 (0.48-0.88)	**0.006**
Age				
<65	1		1	
≥65	1.23 (0.96-1.58)	0.107	1.20 (0.87-1.65)	0.261
Gender				
Male	1		1	
Female	0.97 (0.74-1.27)	0.801	0.77 (0.54-1.09)	0.140
BMI				
<24	1		1	
24-27.9	0.92 (0.71-1.19)	0.518	0.91 (0.66-1.26)	0.575
≥28	1.08 (0.71-1.69)	0.691	1.05 (0.63-1.75)	0.857
Smoking				
No	1		1	
Yes	1.14 (0.88-1.47)	0.317	1.18 (0.86-1.62)	0.305
ECOG				
<2	1		1	
≥2	1.49 (0.93-2.37)	0.094	1.14 (0.64-2.03)	0.660
Tumor type				
NSCLC	1		1	
SCLC	1.33 (0.99-1.78)	0.058	2.19 (1.56-3.09)	**<0.001**
Line of immunotherapy				
1	1		1	
≥2	1.14 (0.88-1.47)	**<0.001**	1.22 (0.89-1.65)	0.214

irTD, immune-related thyroid dysfunction; HR, Hazard Ratio.

The bolded values indicate that the data are statistically significant (p < 0.05).

**Figure 5:**
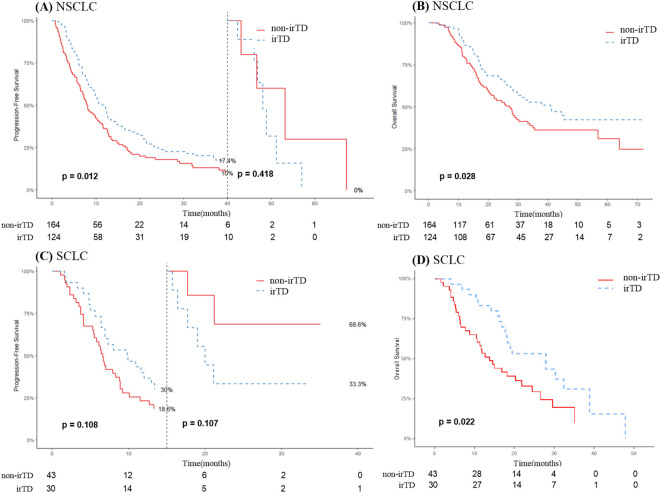
PFS **(A)** and OS **(B)** curves of patients with NSCLC with irTD vs non-irTD groups. PFS **(C)** and OS **(D)** curves of patients with SCLC with irTD vs non-irTD groups.

At the time of diagnosis, 73 individuals had SCLC, and 288 had NSCLC. For NSCLC patients who received immunotherapy and subsequently developed irTD, there was a significant improvement regarding both PFS and OS. Specifically, the median PFS (mPFS) was 11.7 months for the irTD group compared to 8.0 months for the non-irTD group (**Figure 5A**). The median OS (mOS) was also prolonged, at 40.8 months for the irTD group versus 27.2 months for the non-irTD group (HR: 0.68, 95% CI: 0.48-0.96; P = 0.028) (**Figure 5B**). However, among SCLC patients, the difference was only significant for mOS; the irTD group had a mOS of 27.9 months, while the non-irTD group had a mOS of 13.8 months (HR: 0.51, 95% CI: 0.29-0.90; P = 0.022) (**Figure 5D**). In SCLC patients without progression risk, irTD appears to have reduced efficacy before and after 15 months (**Figure 5C**).

**Figure 6:**
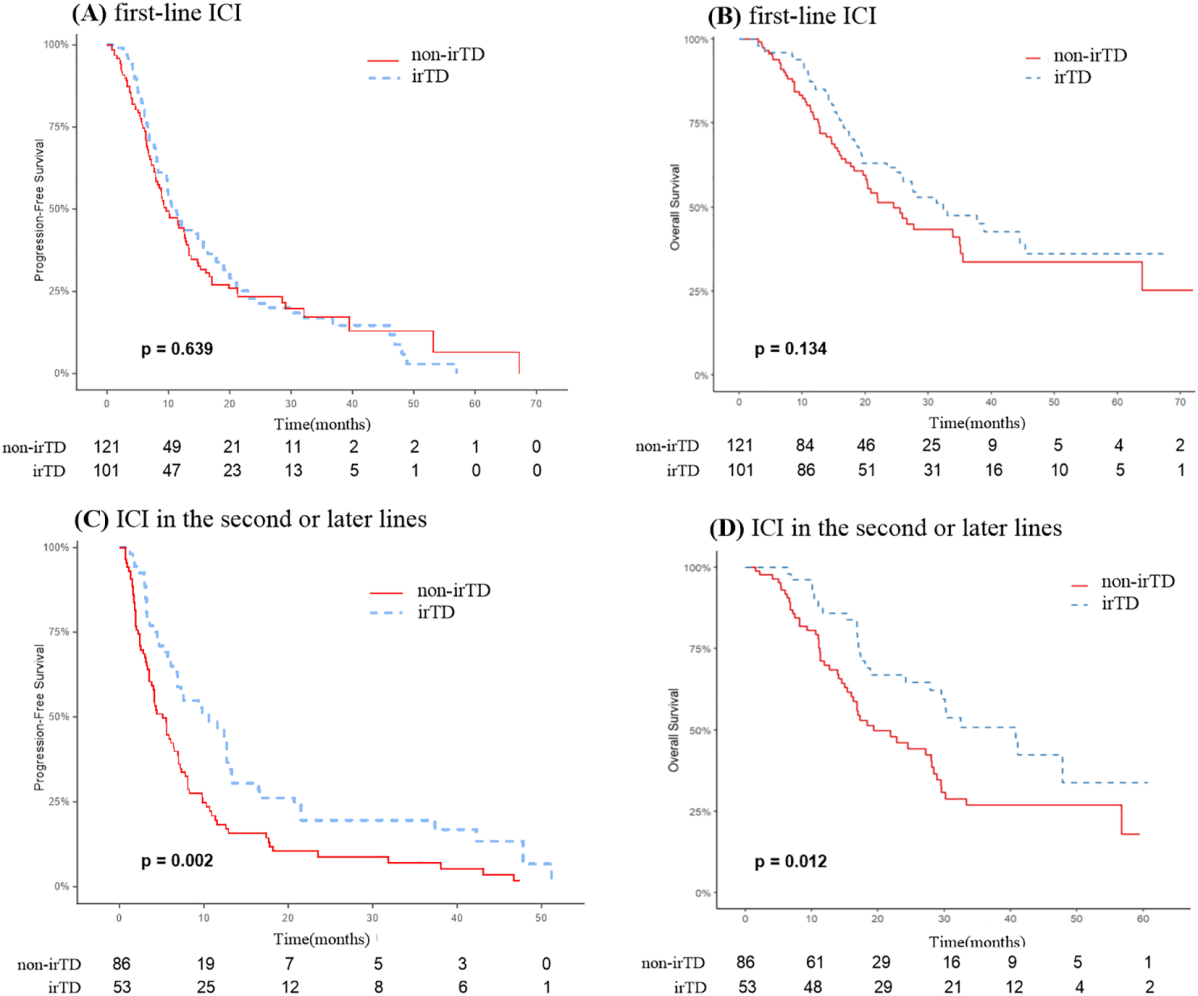
PFS **(A)** and OS **(B)** curves of patients receiving immunotherapy in the first line with irTD vs non-irTD groups. PFS **(C)** and OS **(D)** curves of patients receiving immunotherapy in the second or later lines with irTD vs non-irTD groups.

Among the 222 patients who received immunotherapy as their first-line treatment, PFS and OS did not significantly differ between those with and without irTD. The irTD group’s mPFS was 10.9 months, compared to 9.6 months for the non-irTD group (HR: 0.93, 95% CI: 0.68-1.27; P = 0.639) (**Figure 6A**). The irTD group’s mOS was 32.4 months, while the non-irTD group’s mOS was 24.5 months (HR: 0.74, 95% CI: 0.50-1.10; P = 0.134) (**Figure 6B**). Conversely, for the 139 patients who underwent immunotherapy as a second-line or subsequent treatment, PFS and OS were significantly longer in those who developed irTD. The irTD group’s mPFS was 10.6 months, compared to 5.1 months for the non-irTD group (HR: 0.55, 95% CI: 0.39-0.80; P = 0.002) (**Figure 6C**). The mOS for the irTD group was 40.8 months, while for the non-irTD group, it was 19.4 months (HR: 0.56, 95% CI: 0.35-0.88; P = 0.012) (**Figure 6D**).

None of the patients experiencing irTD exhibited side effects of grade 3 or higher, and no discontinuation of immunotherapy was necessary due to irTD. Furthermore, statistical analysis revealed no significant differences in outcomes based on irTD severity or type. This included comparisons between grade 1 and grade 2 irTD, clinical versus subclinical irTD, and isolated thyrotoxicosis versus isolated hypothyroidism and hypothyroidism with transient thyrotoxicosis (P > 0.05) (**Table 3**).

**Table 3:** The impact of different severities, clinical presentations, and types of irTD on PFS and OS.

Variables	Progression-free Survival			Overall Survival		
	mPFS, months	P	HR (95CI)	mOS, months	P	HR (95CI)
Grade						
G1	11.6	0.954	1	30.2	0.197	1
G2	10.4		1.14 (0.75-1.72)	47.8		0.69 (0.41-1.14)
Clinical Presentation						
Subclinical irTD	10.9	0.866	1	29.6	0.053	1
Overt irTD	10.5		1.03 (0.71-1.50)	45.4		0.63 (0.39-1.00)
Type						
Isolated thyrotoxicosis	10.0	0.550	0.88 (0.59-1.33)	28.3	0.580	1.16 (0.68-1.99)
Isolated hypothyroidism	12.4	0.810	0.96 (0.66-1.38)	30.3	0.491	1.18 (0.74-1.89)
hypothyroidism with transient thyrotoxicosis	9.9	0.273	1.34 (0.80-2.25)	NR	0.143	0.64 (0.36-1.16)

mPFS, the median of Progression-free Survival; mOS, the median of Overall Survival; irTD, immune-related thyroid dysfunction; HR, Hazard Ratio.

### 3.4 Correlation between objective response rate and irTD

**Figure 7:**
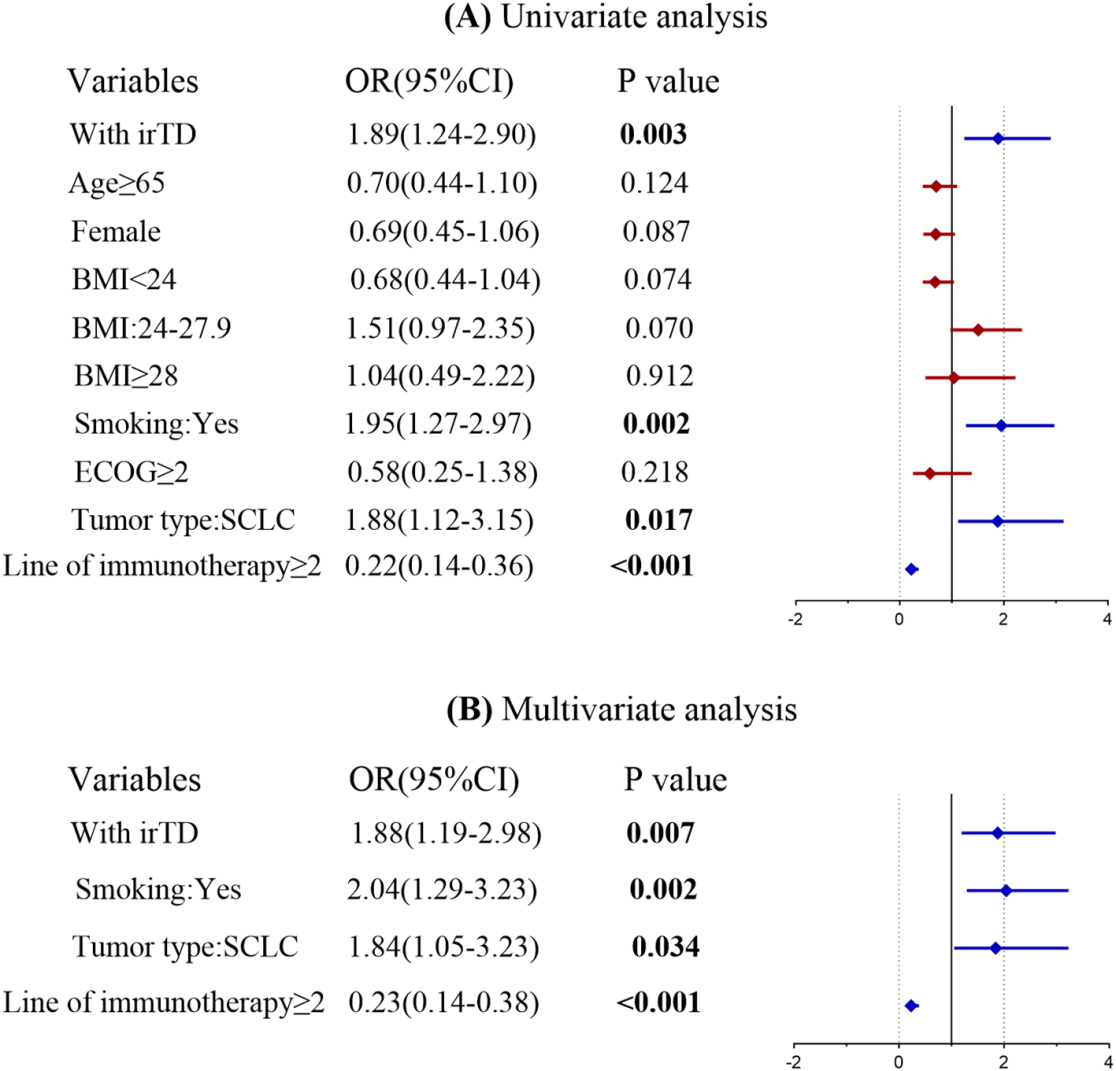
Univariate and multivariate logistic regression forest map of tumor objective response.

A univariate logistic regression analysis was performed to identify potential risk factors associated with achieving an objective tumor response following immunotherapy. The investigation revealed that the occurrence of irTD, smoking history, tumor type, and the number of immunotherapy lines significantly influenced tumor response. Factors from the univariate analysis that yielded significant results were subsequently incorporated into a multivariate logistic regression model. The multivariate analysis demonstrated that the occurrence of irTD (OR = 1.88, 95% CI: 1.19-2.98, P = 0.007) served as a predictor-marker of objective tumor response (**Figure 7**). This outcome underscores the potential of irTD as a reliable marker for predicting treatment outcomes, as it provides a substantial indication of a favorable response to immunotherapy.

### 3.5 Exploratory analysis of potential clinical features in patients with prolonged survival

**Figure 8:**
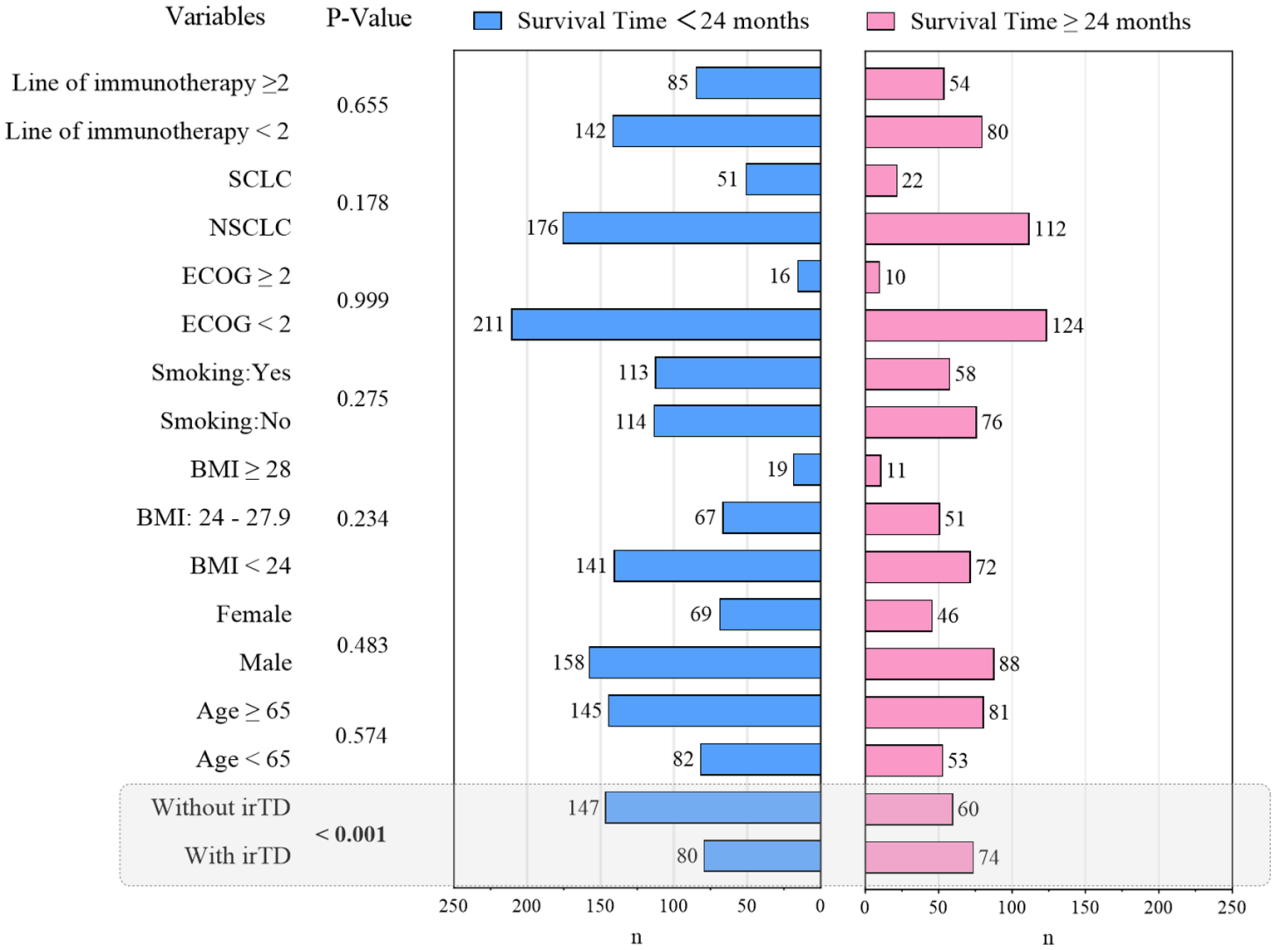
Baseline characteristics of different survival times of patients.

This investigation revealed that patients with advanced or metastatic lung cancer demonstrated one-year and two-year OS rates of 67.0% and 37.1%, respectively, following immunotherapy. To elucidate the clinical characteristics associated with extended survival, a Chi-square analysis was conducted, comparing two groups based on whether survival time post-immune checkpoint inhibitor treatment exceeded 24 months. The results are presented in **Figure 8**. Notably, patients experiencing irTD exhibited a tendency towards prolonged survival (P < 0.001).

## 4 Discussion

The therapeutic efficacy of ICIs in treating advanced cancers is paramount. While ICIs demonstrate significant potential, they may induce immune-related adverse events (irAEs) affecting various systems, including pulmonary, cardiovascular, and digestive (13). Although most irAEs are self-limiting, they can potentially impact the course and effectiveness of immunotherapy, as well as overall patient survival (14). irTD stands as one of the most common endocrine-related adverse events. irTD typically manifests as thyrotoxicosis or hypothyroidism, with the latter occurring more frequently (15).

The frequency of irTD observed in this study, at 42.7%, is consistent with findings from the two largest observational studies on the condition (10, 16). The occurrence of irTD may be attributed to various mechanisms, including T-cell-mediated destructive thyroiditis, autoantibody-mediated thyroid autoimmunity, and a decrease in immunosuppressive mononuclear cells (5, 17, 18). Factors such as BMI, baseline TSH levels, and hypertension have been identified as potential contributors to irTD development (8, 19–24). Significant differences were observed in TSH levels, thyroid autoantibody positivity rates, monocyte counts, NLR, and MLR between the irTD and non-irTD groups. Notably, patients with isolated hypothyroidism demonstrated distinct biological characteristics, particularly regarding TSH levels. Additionally, patients with hypothyroidism who experienced transient thyrotoxicosis exhibited a significantly earlier onset compared to other forms of irTD. These findings indicate that different forms of irTD manifest with distinct clinical characteristics and biological markers.

Our research indicates no significant difference in survival advantage among patients with various types of irTD. This finding contrasts with existing literature, highlighting the ongoing debate in this field. Baek HS et al. reported that patients with newly diagnosed overt or subclinical hypothyroidism demonstrated a significantly reduced risk of mortality (risk ratio: 0.324, P = 0.002) (25). Conversely, Muir et al. observed that overt thyrotoxicosis was associated with improved PFS (HR = 0.68, 95% CI = 0.49–0.94, P = 0.02) and OS (HR = 0.57, 95% CI = 0.39–0.84, P = 0.005) (10). Zhou et al. noted a trend towards improved OS and PFS in their subgroup analysis of hypothyroidism and hyperthyroidism, although it did not reach statistical significance (26). These conflicting results underscore the ongoing uncertainty regarding the specific impact of different irTD forms on survival outcomes. Our study contributes valuable insights to this ongoing scientific discourse.

This study represents the first large-scale retrospective investigation of irTD, in both NSCLC and SCLC concurrently. Previous studies have predominantly focused on patients with NSCLC, with limited consideration of how irTD affects survival outcomes in SCLC patients. Our findings indicate that patients who developed irTD exhibited favorable survival prognoses and treatment response outcomes, aligning with results from several comprehensive retrospective studies (8, 26–30). However, it is important to note that this correlation may be influenced by additional factors.

In our research, we noted a markedly reduced NLR and MLR in the irTD cohort when compared to the non-irTD cohort. Recent findings from the IMpower133 trial further emphasized a significant association between elevated Tumor Mutational Burden (TMB) adjusted for low NLR and enhanced OS in SCLC patients receiving atezolizumab (P=0.001) or placebo (P=0.034) (31). Moreover, a substantial retrospective analysis involving patients with advanced or metastatic melanoma, NSCLC, and Renal Cell Carcinoma (RCC) revealed a strong correlation between diminished OS and heightened MLR (32). These results are consistent with our observations, indicating that low NLR and MLR may play a role in the development of irTD, and that patients exhibiting irTD experience a more favorable prognosis.

To further investigate the factors influencing these outcomes, we conducted a subgroup analysis with patients experiencing different pathological categories of lung cancer. The occurrence of irTD was significantly associated with improved PFS and OS in patients with NSCLC. This observation aligns with several previous studies focusing exclusively on patients with NSCLC. For example, one study found that, compared to the non-irTD group, patients with irTD caused by nivolumab had a significantly longer mOS (16.1 vs. 13.6 months, HR 0.61; 95% CI 0.39-0.93) (33). Similarly, Iwama et al. demonstrated that thyroid irAE was associated with significantly longer survival for individuals with NSCLC (34). A meta-analysis of 11 studies involving 1,962 patients with NSCLC revealed that patients who developed irTD also exhibited improved PFS (HR 0.54, 95% CI: 0.44-0.64) and OS (HR 0.34, 95% CI: 0.25-0.44) (29). While these data provide compelling evidence of a relationship between irTD and improved survival outcomes for individuals with NSCLC, large-scale and multicenter prospective trials are necessary to confirm these findings. PFS in patients with SCLC did not differ statistically significantly. However, consistent with Zhang et al.’s findings, the mOS for patients in the irTD group was significantly longer than that of the non-irTD group. They identified irTD as a crucial prognostic indicator for patients with stage IV SCLC, significantly correlating with improved outcomes (35). The absence of improvement in PFS in irTD patients may be attributed to the short duration of the immunotherapeutic response observed in most patients with SCLC. Approximately 15% of all lung tumors are classified as SCLC, characterized by a high risk of recurrence, early metastasis, and a poor prognosis (36). Although nearly all SCLC patients respond effectively to early-stage treatment, resulting in a significant reduction in tumor size, they are highly prone to recurrence (37). The impact of treatment is substantially reduced after recurrence. Its distinct tumoral heterogeneity is closely associated with tumor evolution, metastasis, and acquired drug resistance (38). While ICIs offer a promising new approach for treating patients with SCLC, only a subset has experienced prolonged survival benefits. The identification of irTD as a potential prognostic biomarker warrants further investigation.

To investigate the predictive significance of irTD, we conducted an exploratory study on patients receiving various lines of immunotherapy. Our results revealed variations in the association between irTD and survival prognosis based on whether immunotherapy was administered as a first-line treatment or as a second-line or subsequent treatment. Notably, significantly prolonged PFS and OS were associated with irTD exclusively in patients who received immunotherapy as a second-line or later therapeutic approach, not observed in first-line treatment. We performed a comprehensive analysis of the clinical characteristics of patients in the irTD and non-irTD groups across different treatment lines to identify potential factors influencing irTD’s predictive capacity for survival. Among first-line immunotherapy patients, we observed a higher proportion of stage IIIB patients without irTD and a higher proportion of patients without brain metastases (BMS) compared to those with irTD (stage IIIB: 19.0% vs 15.8%; No brain metastases: 88.4% vs 86.1%). Given that stage and brain metastasis are crucial factors affecting lung cancer treatment efficacy, and the prognosis for patients with BMS is extremely poor, with an average survival time of only 1 to 2 months for untreated patients (39), this may have partially obscured the impact of irTD on the prognosis of this population, resulting in no significant difference in PFS and OS in the non-irTD group. Conversely, among patients receiving second-line immunotherapy and beyond, the proportion of patients without BMS was lower in the group without irTD compared to the group with irTD (74.7% vs 79.2%). This disparity may have further accentuated the prognostic difference between the two groups, leading to statistically significant results. Although our exploratory analysis suggests that irTD may have some predictive value in second-line and subsequent therapy, these findings require validation through further studies with expanded sample sizes due to current limitations. Furthermore, the tumor microenvironment and immune system changes in patients treated with second-line and subsequent therapies are complex. While irTD may offer some predictive value, clinicians must consider the patient’s overall condition and treatment context when making decisions.

The exceptional efficacy of immune checkpoint inhibitors in lung cancer has renewed patient optimism for long-term survival. Several researchers (40, 41) have begun investigating the relationship between clinical characteristics and long-term survival in lung cancer patients. This study similarly examined the association of clinical features with long-term survivors (LTS), defined as patients who survived beyond 24 months after immune checkpoint inhibitor treatment. The findings revealed that patients who developed irTD following immuno-combination therapy demonstrated a higher likelihood of becoming LTS (P < 0.001).

This study has several limitations. First, despite efforts to control for confounding variables, complete accounting for potential confounders was not achievable. Second, the trial exclusively included patients with locally progressed or advanced lung cancer; the impact of irTD on the short- and long-term prognoses of patients receiving neoadjuvant immunotherapy remained unexamined. Third, thyroid peroxidase antibody (TPOAb) is one of the most commonly detected autoantibodies in thyroid disease. However, due to the lack of routine testing for TPOAb in the hospital, TPOAb levels of patients were not obtainable. Finally, due to the varying frequency of thyroid hormone testing and the inconsistent intervals between retests for each patient, comprehensive data on changes in thyroid hormone levels could not be collected. While this study offers preliminary insights into irTD, its limitations underscore the need for future research. Enhancing study design and methodology is imperative to elucidate the mechanism of irTD and its clinical impact more comprehensively and accurately, thereby providing more substantial support for clinical practice.

## 5 Conclusion

In conclusion, irTD demonstrates potential as a predictive indicator for long-term survival benefits in Chinese patients with lung cancer. This observation holds true for both NSCLC and SCLC patients. However, it is important to note that irTD exhibited predictive value specifically for individuals receiving immunotherapy as a subsequent line of treatment. Although considerable heterogeneity exists among different types of irTD, their impact on survival prognosis remains largely consistent. Further research is necessary to validate the reliability of irTD as a prognostic marker, with the ultimate goal of maximizing survival benefits for patients.

## Data Availability

The original contributions presented in the study are included in the article/**Supplementary Material**. Further inquiries can be directed to the corresponding author.
